# Development and Evaluation of a New Lateral Flow Immunoassay for Serodiagnosis of Human Fasciolosis

**DOI:** 10.1371/journal.pntd.0001376

**Published:** 2011-11-08

**Authors:** Victoria Martínez-Sernández, Laura Muiño, María Jesús Perteguer, Teresa Gárate, Mercedes Mezo, Marta González-Warleta, Antonio Muro, José Manuel Correia da Costa, Fernanda Romarís, Florencio M. Ubeira

**Affiliations:** 1 Laboratorio de Parasitología, Facultad de Farmacia, Universidad de Santiago de Compostela, Santiago de Compostela, Spain; 2 Laboratorio de Parasitología, Centro Nacional de Microbiología, Instituto de Salud Carlos III, Madrid, Spain; 3 Laboratorio de Parasitología, Centro de Investigaciones Agrarias de Mabegondo, INGACAL, Abegondo, A Coruña, Spain; 4 Laboratorio de Inmunología y Parasitología Molecular, CIETUS, Facultad de Farmacia, Universidad de Salamanca, Salamanca, Spain; 5 Departamento de Doenças Infecciosas, CSPGF-INSA, Porto, Portugal; McGill University, Canada

## Abstract

**Background:**

Human fasciolosis is a re-emerging disease worldwide and is caused by species of the genus *Fasciola* (*F. hepatica* and *F. gigantica*). Human fasciolosis can be diagnosed by classical coprological techniques, such as the Kato-Katz test, to reveal parasite eggs in faeces. However, although 100% specific, these methods are generally not adequate for detection of acute infections, ectopic infections, or infections with low number of parasites. In such cases immunological methods may be a good alternative and are recommended for use in major hospitals where trained personnel are available, although they are not usually implemented for individual testing.

**Methodology/Principal Findings:**

We have developed a new lateral flow test (SeroFluke) for the serodiagnosis of human fasciolosis. The new test was constructed with a recombinant cathepsin L1 from *F. hepatica*, and uses protein A and mAb MM3 as detector reagents in the test and control lines, respectively. In comparison with an ELISA test (MM3-SERO) the SeroFluke test showed maximal specificity and sensitivity and can be used with serum or whole blood samples.

**Conclusions/Significance:**

The new test can be used in major hospitals in hypoendemic countries as well as in endemic/hyperendemic regions where point-of-care testing is required.

## Author Summary

Fasciolosis is an important plant-borne trematode zoonosis. This disease is of both clinical and veterinary relevance and, according to the WHO, is considered a re-emerging disease that is spreading around the world. Fasciolosis has a serious impact on health because of the large size of the parasite and the effects of the parasite in down-regulating the host immune response. Human fasciolosis can be distinguished by an acute phase, in which the parasite migrates through different tissues, and a chronic phase in which it invades the bile ducts. Here we describe the development of a rapid, simple and inexpensive immunochromatographic diagnostic method, based on the use of a recombinant cathepsin L1 protein, which performs better than other more complex indirect methods, providing similar specificity and higher sensitivity. The simplicity of the method represents a great advantage for the intervention systems applied in different endemic areas by WHO, such as passive case finding (e.g. Vietnam) and selective treatment (e.g. Egypt). Because of its characteristics, the system can be applied to both phases of the disease, and in holo, meso and hyperendemic areas where point-of-care testing is required.

## Introduction

Fascioliosis ( = fascioliasis) is a plant-borne zoonosis caused by infection with trematode species of the genus *Fasciola*. Human fasciolosis is a re-emerging disease present worldwide, and can be produced by *Fasciola hepatica* and *Fasciola gigantica*. In Europe, the Americas and Oceania, only *F. hepatica* is present, but in several areas of Africa and Asia the geographical distribution of both species may overlap [1] and even hybridize in some cases [2], [3]. Overall, it is considered that between 2.4 [4] and 17 million [5] people from 55 countries are infected by *Fasciola* species and that 180 million people are at risk of infection [1]. Fasciolosis is an important health problem in some South American countries (Bolivia, Peru, Chile and Ecuador), in the Caribbean (Cuba), northern Egypt, and in the Caspian region (Iran), although human infections by *Fasciola* are not infrequent in European countries such as Portugal, Spain, France and the United Kingdom [6]. Furthermore, it is expected that this trematode-induced disease will increase over time as a result of climate change and the advent of milder, wetter weather [7], [8].

Human fasciolosis can be diagnosed by classical coprological techniques, such as the Kato-Katz test [9], to reveal parasite eggs in faeces. However, although 100% specific, these methods have some limitations including: i) non usefulness during the prepatent period (first 3–4 months after infection [10]); ii) poor sensitivity in patients with a low degree of parasitization, or in patients with intermittent egg shedding; iii) non usefulness in ectopic infections or when parasites do not reach maturity.

To avoid the above problems, some immunological techniques based on the determination of circulating secretory antigens [11], [12], testing of coproantigens [11], [13], or determination of serum anti-*Fasciola* antibodies [14]–[18] have been reported in the last two decades. However, for detection of early stages of *Fasciola* infections in humans, or ectopic infections, antibody determinations are preferable to coprological tests as circulating antibodies are produced early on and remain detectable for long periods.

Several antigenic fractions of *Fasciola* [19], [20], purified antigens [21], [22] and recombinant antigens [15], [23], [24] have been successfully used for the serodiagnosis of fasciolosis in human and animal species. Nevertheless, cathepsins L are the most frequently used target antigens for detecting anti-*Fasciola* antibodies [15], [21], [25], [26], as circulating antibodies to these molecules remain at high levels for long periods [27]. An ELISA test (MM3-SERO) that had proven useful for serodiagnosis of fasciolosis in several animal species with maximal sensitivity and specificity [27]–[30], was recently found to involve binding to cathepsins L1 and L2 from both species of *Fasciola* [31].

Despite the usefulness of serological ELISAs for the diagnosis of fasciolosis, human infections frequently occur in non-developed countries where access to diagnostic laboratories is not always possible. In this sense, the development of rapid lateral flow immunoassays (LFIAs), also known as immunochromatographic tests, would be of great interest. In this study we report the development and evaluation of a new LFIA for serodiagnosis of human fasciolosis, which can be used for point-of-care (POC) testing.

## Materials and Methods

### Ethics statement

The study protocol was approved by the Ethics Committee of the Universidad de Santiago de Compostela, Spain. The serum samples used in this study were obtained as part of public health diagnostic activities, were already collected before the start of the study, and were tested as anonymous samples. Control (negative) blood samples were obtained from volunteers after they had provided written informed consent.

### Human sera and blood samples

The serum samples (n = 203) used in this study were obtained from serum collections stored in the Centro Nacional de Microbiología (ISCIII, Madrid, Spain), Laboratorio de Parasitología (Facultad de Farmacia, USC, Spain), Centro de Investigación de Enfermedades Tropicales de la Universidad de Salamanca (CIETUS, Salamanca, Spain) and the Centre for Parasite Immunology and Biology (CSPGF-INSA, Porto, Portugal). We analyzed serum samples from 39 patients with fasciolosis, 27 patients with schistosomosis, 20 patients with filariosis, 9 patients with hydatidosis, 15 patients with anisakiosis, 22 patients with toxocariosis, 12 patients with Chagas' disease, and sera from 59 patients with non-infectious pathology (mainly from surgical interventions for non-painful processes such as cataracts and hernias). The above infections were diagnosed in the country of origin of the patients by one or more of the following methods: i) routine coprological techniques for detection of ova and parasites (Kato-Katz and Ritchie tests, using 3 stool samples), (ii) examination of urine for *Schistosoma haematobium* eggs, after sedimentation, (iii) Knott's test for detection of microfilaremia in blood, (iv) the ICT Filariasis test (Binax, Portland and Maine) for detection of *Wuchereria bancrofti*, (v) an “in house” ELISA test with soluble antigens present in sheep hydatidic cysts as target [32], [33], (vi) detection of IgE antibodies to the allergens Ani s 1 and Ani s 7 from *Anisakis* [34], (vii) Enzymun-test IgE (Boehringer-Mannheim Lab., Mannheim, Germany) for detection of anti-*Toxocara* antibodies in serum, and (viii) the Chagatest-ELISA recombinante (Wiener Laboratorios S.A.I.C., Rosario, Argentina) for detection of serum antibodies in Chagas' disease.

*Fasciola* seropositive patients from Portugal were tested by using *Fasciola* excretory-secretory antigens (ESAs) as target in ELISA and/or by Western-blotting analysis [35], while seropositive patients from Salamanca were tested by an ELISA test with *Fasciola* ESAs, according to Hillyer et al. [36], and patients from Madrid were diagnosed by use of a haemagglutination assay (Fumouze Diagnostics, Paris, France). All *Fasciola* positive patients from Santiago de Compostela, and the above positive sera were confirmed for the presence of anti-*Fasciola* antibodies by use of the MM3-SERO ELISA (see below).

Negative whole blood samples were obtained from 12 volunteers (8 women and 4 men, aged 20–55 years) by fingertip puncture with a lancet (Glucoject, Menarini Diagnostics, Firenzi, Italy), at the Faculty of Pharmacy, University of Santiago de Compostela, Spain.

### Cloning of a procathepsin L1 gene (rpCL1) from adult *Fasciola hepatica*

An L1-cathepsin from *F. hepatica* (gb|FR848428) was cloned as previously reported [31]. Briefly, the encoding gene without the putative N-terminal fragment (signal peptide) was cloned into the pQE expression vector (QIAGEN, QIAGEN Iberia S.L., Madrid) with the primers 5′-pCL1 TCGAATGATGATTTGTGGCATCAGTGGAAGCG (forward) and 3′-pCL1 CGGAAATCGTGCCACCATCGG (reverse), and further transformed into the M15 [pREP4] strain of *E. coli* (QIAGEN, [37]). *Fasciola hepatica* recombinant rpCL1 expression was induced by addition of 1 mM IPTG.

### Purification and refolding of the rpCL1

After induction, cells were harvested by centrifugation and the insoluble recombinant proteins were purified with B-PER reagent (Thermo Fisher), solubilized and purified by affinity-chromatography with HIS-Select Nickel Affinity Gel (Sigma-Aldrich) under denaturing conditions, as indicated by the supplier (8 M urea). The protein solution containing the rpCL1 was then refolded by dispersing the eluate at a ratio of 1∶ 50 in PBS containing cysteine (10 mM) and cystine (1 mM), followed by membrane-filtration concentration in an Amicon Stirred Ultrafiltration Cell equipped with a Filtron Omega Series membrane (10 K nominal molecular weight limit; Pall Filtron Corporation). Finally, the protein concentration was measured with the Micro BCA Protein Assay Kit (Pierce, Rockford, IL), adjusted to 2 mg/ml in PBS, and the sample was stored at −80°C until use.

### MM3 ELISA determinations

Human serum samples were analyzed by MM3-SERO ELISA, a capture immunoassay that detects antibodies against the highly specific antigens recognized by mAb MM3 [29]. Polystyrene microtitre F16 plates (Greiner Bio-One, Sigma-Aldrich, Madrid, Spain) were coated for 2 h at 37°C with mAb MM3 (100 µl/well of a solution containing 5 µg/ml protein in PBS), and the uncoated sites were blocked with a 1.5% solution of buffered sodium caseinate for 1 h at room temperature (RT). After washing once with PBS containing 0.2% Tween-20 (PBS-T), 100 µl of either *F. hepatica* ESAs (0.4 µg/ml total protein) in PBS-T, or 100 µl of PBS only, were added to odd (Ag+) and even (Ag−) plate rows, respectively, and then incubated for 1 h at RT. After washing 4 times with PBS-T, 100 µl of each serum sample, diluted 1/100 in PBS-T containing 1% of skimmed milk, were added to each Ag+ and Ag− ELISA well, in duplicate. Plates were then incubated for 2 h at 37°C, washed 5 times with PBS-T, and incubated at 37°C for 1 h with 100 µl/well of anti-human IgG1 monoclonal antibody (Sigma-Aldrich) labelled with FITC and diluted 1/3000. The reaction was revealed with peroxidase-conjugated rabbit anti-FITC IgG (Serotec; dilution 1/5000). Plates were washed again 5 times with PBS-T, and 100 µl of substrate (SigmaFast OPD, Sigma-Aldrich) were added to each well. The plates were then incubated for 20 minutes, and the reaction was stopped with 25 µl of 3N H_2_SO_4_. The optical density (OD) was measured at 492 nm in an ELISA reader. The OD value for each sample was calculated as OD1 minus OD2, where OD1 is the mean for the 2 Ag+ wells, and OD2 is the mean for the 2 Ag− wells. A cut-off value of OD = 0.05 for positive responses was calculated as the OD mean plus four times the standard deviation obtained for 200 human serum samples (age range: 20–76 years) from patients with eosinophilia of unknown origin but negative for *Fasciola*, by use of the Fumouze's haemagglutination test. However, to avoid false positive reactions due to batch variations in secondary ELISA reagents, a doubtful range (OD = 0.05–0.099) was arbitrarily established for this test, while OD values equal or higher than OD = 0.1 were considered positive.

### LFIA development

#### Preparation of colloidal gold conjugate

The colloidal gold conjugate used in this study was prepared with the Gold in a Box conjugation kit (BioAssay Works, Ijamsville, MD), according to the supplier's instructions. Briefly, 0.5 ml samples of the colloidal gold solution (15 OD, 40 nm) buffered in the pH range 7–10 were mixed with the solution of *Fasciola* rpCL1 (28 µg/ml) and maintained at RT with some additional mixing for 30 min. Each solution was then tested for stability by mixing 10 µl of 1 M NaCl with the same amount of gold conjugate. Once the desired pH was selected, a batch of 40 ml gold conjugate was prepared, with the same concentration of rpCL1 as above. The conjugate was then stabilized with the Blocking Stabilizer Solution (provided by the manufacturer) and allowed to stand at RT for a further 16 h. Finally, the gold conjugate was used to construct the LFIA device.

#### Preparation of the LFIA strips

**Figure 1:**
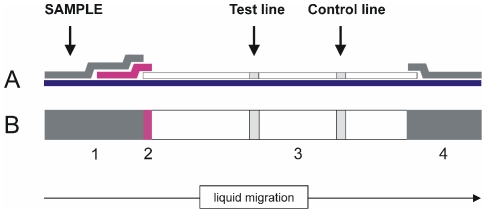
Schematic view of the SeroFluke device. A) lateral view, B) front view. The strip components are: 1) sample pad, 2) conjugate pad, with rpCL1 coupled to colloidal gold, 3) nitrocellulose membrane, 4) wick. The test and control lines contain recombinant *Staphylococcus* protein A and mAb MM3, respectively.

The LFIA device (Fig. 1) was composed of a sample pad of cellulose fibre (CFSP203000, Millipore Iberica SA, Barcelona, Spain), a glass fibre conjugate pad (GFCP103000 Millipore), a nitrocellulose (NC) membrane on a plastic back (HF135MC100, Millipore) and an absorbent cellulose fibre pad (CFSP173000, Millipore). A solution of 1 mg/ml of recombinant *Staphylococcus* Protein-A (Sigma) and a solution of 1 mg/ml of mAb MM3, both in PBS, were dispensed in the detection and control lines of the NC membrane, respectively, using a BioDot XYZ platform (BioDot Inc., Irvine, CA) at 1 µl/cm. After the reagents were dispensed, the NC membranes were dried overnight at 37°C.

The glass fibre containing the gold conjugate was prepared by immersion of the conjugate pads in the colloidal solution and the pads were then dried overnight at 37°C on a stainless steel mesh. Finally, the sample pad, conjugate pad and the absorbent pad were sequentially assembled on the NC membrane cards with a 2 mm overlap. Finally, the assembled plates were cut into 3-mm-wide strips with a manual 550-AP Kobra Cut guillotine (Elcoman SLR, Milan, Italy) and stored in the presence of a desiccant, at 4°C, before use.

#### Test procedure

To test for anti-*Fasciola* IgG antibodies in serum, the sera previously diluted 1/100 in 200 µl of SeroFluke buffer were placed in the wells of a polystyrene microtitre plate to which the LFIA strips were applied and allowed to develop for 10 min. During the assay, the antibodies present in the sample migrate with the buffer through the device and bind to the rpCL1-gold conjugate forming antibody-rpCL1-gold (ACG) complexes, which continue migrating through the NC membrane. In positive samples, most of the ACG complexes are retained by protein A in the NC test line producing a coloured band. The excess rpCL1-gold conjugate with free MM3-recognizing epitopes is not retained in the test line, and continues to migrate until reacting with MM3 mAb, which is present in the control line of the NC membrane, thus producing a second coloured band. In negative samples, the rpCL1-gold conjugate is not retained in the test line because the ACG complexes are not formed, thus producing a single coloured band (control line).

For determination of anti-*Fasciola* antibodies in whole blood samples, each sample (10 µl plus 2 µl of positive serum, or 10 µl of blood alone) was mixed with 190 µl of SeroFluke buffer in the microtitre well and allowed to haemolyze for 2 min. The LFIA strips were then placed in each sample and allowed to react for 10 min as above. The strips were then cut transversally with scissors at the starting point of the NC on each strip, and placed in an another microtitre well containing 200 µl of SeroFluke buffer for washing. After 10 min the results were read as for the serum samples.

**Figure 2:**
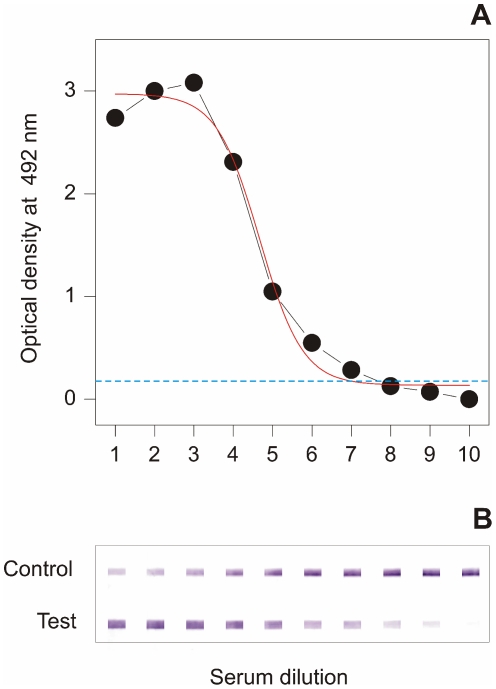
Comparison of the detection limit of the MM3-SERO and SeroFluke tests. The ability of MM3-SERO ELISA (A) and SeroFluke (B) to detect anti-*Fasciola* antibodies in two fold dilutions of pooled positive sera was compared. The starting serum dilution was 1/100 for both tests.

For comparison with MM3-ELISA OD values, the signals obtained with positive sera using the SeroFluke test were classified in arbitrary units (AUs) on a scale of 0 (negative) to 5 (strongly positive), according to the intensity of the signal obtained in the test and control lines of the device. The AU colour scale was created according to the signals obtained with the pooled sera described in Fig. 2. The following equivalence was used: AU = 0 (negative); AU = 1 (as for serum dilution 9); AU = 2 (as for serum dilutions 7 and 8); AU = 3 (as for serum dilutions 5 and 6); AU = 4 (as for serum dilutions 3 and 4; and AU = 5 (as for serum dilution 1 or lower).

## Results

### Design and use of the SeroFluke test

**Figure 3:**
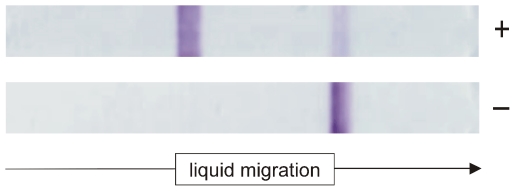
A typical positive and negative result with the SeroFluke test. A strong positive signal in the test line with the positive sample, and no signal with the negative serum can be observed.

The HF135 (Millipore) NC membranes, which allow flow rates of 135±34 sec/4 cms, a pad conjugate containing rpCL1 bound to colloidal gold at pH 8.6, together with protein A in the test line, and mAb MM3 in the control line, were used to construct a lateral diffusion test for serodiagnosis of human fasciolosis. Typical results for a positive and a negative serum are shown in Fig. 3. As can be observed, both positive and negative lines were strongly coloured, and therefore suitable for screening with the naked eye. The data in Fig. 3 also show that negative samples produced a strong signal in the control line with no background in the test zone.

To investigate the detection limit of the assay [38], we compared the signals obtained with two-fold serial dilutions of three positive pooled sera in the LFIA, with those obtained in the MM3-ELISA used as gold standard. The data in Fig. 2 show that the new LFIA is highly sensitive, as it was able to recognize as positive the pooled sera at a higher dilution (dilution 9; 1/25600) than the MM3-SERO test (dilution 7; 1/6400). With respect to the stability of the SeroFluke test, we observed that the detection limit did not change after storage for three months at 37°C, or 6 months at room temperature, when the strips were maintained in plastic bottles with a desiccant (data not shown).

### Analysis of the sensitivity and specificity of the SeroFluke test

In order to evaluate the sensitivity and specificity of the new SeroFluke test, we compared the results obtained with this test and the MM3-SERO ELISA test, using sera from patients positive for fasciolosis and sera from patients with the other parasitic infections described above. The data in Table 1 show an almost perfect concordance of results obtained by both methods, for positive (n = 39) and negative samples (n = 164). In fact, the only partial discrepancy was one serum sample containing a very small amount of anti-*Fasciola* antibodies, which tested doubtful (OD = 0.08) in the MM3-SERO ELISA and positive in the SeroFluke test (Table 2). The mean value for the MM3-SERO ELISA in the group of *Fasciola* positive sera was OD = 1.61 (OD range: 0.08–2.71), all of which produced a clear signal in the test line of the SeroFluke device, according to the AUs scale indicated in the Material and Methods section. The data in Table 2 also indicate that although some positive sera produced higher signals with SeroFluke than with MM3-SERO, more than 82% of sera that produced strong signals in the SeroFluke test (ranks 3–5 AU) also produced good signals (>OD = 0.5) in ELISA.

**Table 1:** Reactivity of several types of human sera with *Fasciola hepatica* rpCL1.

Diseases	Sera (n)	MM3-SERO positive	MM3-SERO doubtful	MM3-SERO negative	SeroFluke positive	SeroFluke negative
Fasciolosis	39	38	1	0	39	0
Schistosomosis	27	0	0	27	0	27
Filariosis	20	0	0	20	0	20
Hydatidosis	9	0	0	9	0	9
Anisakiosis	15	0	0	15	0	15
Toxocariosis	22	0	0	22	0	22
Chagas' disease	12	0	0	12	0	12
Other pathologies	59	0	0	59	0	59
**Total**	**203**	**38**	**1**	**164**	**39**	**164**

The reactivity of 203 human sera from patients with several parasitic diseases and other pathologies were compared using the MM3-SERO and SeroFluke tests.

**Table 2:** Reactivity of *Fasciola* positive sera measured with MM3-SERO and SeroFluke tests.

MM3-SEROOD ranks	SeroFluke (AUs)						TOTAL
	0	1	2	3	4	5	
0.050–0.100	0	0	1	0	0	0	1
0.101–0.250	0	0	0	2	1	0	3
0.251–0.500	0	0	0	3	1	0	4
0.501–1.000	0	0	0	4	2	1	7
1.001–2.000	0	0	0	8	5	3	16
2.001–3.000	0	0	0	2	5	1	8
**TOTAL**	**0**	**0**	**1**	**19**	**14**	**5**	**39**

### Usefulness of the SeroFluke test for detection of anti-Fasciola antibodies in whole blood

**Figure 4:**
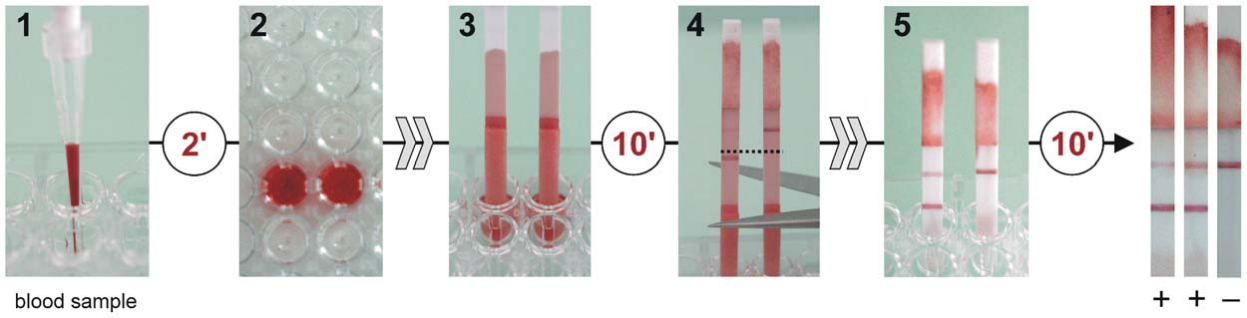
Schematic sequence showing the use of SeroFluke strips with whole blood. The sample (10 µl) was diluted 1/20 with SeroFluke buffer in a polystyrene microtitre plate well and allowed to haemolyze for 2 min (1, 2). The LFIA strip was then placed in the well, and allowed to react for 10 min (3). The strip was then cut at the beginning of the nitrocellulose membrane (4) and transferred to a new well containing 200 µl of fresh SeroFluke buffer (5). The results were read after washing the strip for another 10 min.

Once we had evaluated the usefulness of the SeroFluke test for diagnosing human fasciolosis in sera, we checked the validity of the test for diagnosing human *Fasciola* infection by using whole serum samples. Because of the non availability of whole blood from positive patients, we mixed blood samples from 12 healthy volunteers with pooled positive or negative sera. As indicated above, the test was run for 10 min, and the device was then washed in fresh buffer to remove the haemoglobin, thus facilitating the visualization of positive lines. The practical use of this test with whole blood samples and the results obtained after the washing step for two positive and one negative serum samples are shown in Fig. 4.

## Discussion

The SeroFluke LFIA described in this study is the first one-step LFIA developed for serodiagnosis of human fasciolosis and, to our knowledge, it is also the first LFIA device developed for serodiagnosis of a trematode-induced disease. As reported above, human fasciolosis can also be correctly diagnosed with several ELISA tests [17], [18], [23], or the MM3-SERO described here, but in general these immunoassays must be carried out in specialized centres, by trained personnel. However, the SeroFluke test can be used by personnel with minimal training, and more importantly, it can be used for POC testing, which is of great importance for medical attention of populations in several countries where this trematodosis is endemic [9]. In such countries, the possibility of using whole blood samples for the SeroFluke test (obtained by e.g. taking a drop of blood by fingertip puncture with a lancet, as in this study, or even collected on filter paper [39]) is also advantageous, as this is cheaper and saves the time and expense involved in obtaining serum samples. However, even considering only major laboratories in hypoendemic countries, the new LFIA may offer advantages over ELISA methods, as it allows individual testing of patients, whereas ELISA methods are designed to test several patients at once and some robustness may be lost when they are used only sporadically [40]. Furthermore, the LFIAs can be produced in small quantities (e.g. in the range of 10–25 tests), which would reduce the costs associated with the shelf-life of the kits. In this respect it should be considered that the use of serological methods to detect anti-*Fasciola* antibodies in non-endemic countries is frequently limited to patients with eosinophilia of unknown origin, or to confirm infection suspected after radiological analysis.

In the present study, only one *Fasciola* serum sample tested doubtful by MM3 SERO. The sample was obtained from a patient who had tested positive by MM3 SERO two years before and was subsequently treated with triclabendazole. Assuming such serum to be truly positive, the SeroFluke test displayed 100% sensitivity and 100% specificity. Moreover, as demonstrated by testing serial serum dilutions (Fig. 2), the SeroFluke test showed a lower detection limit, which enables clear positive signals to be obtained with sera from patients with very low circulating anti-*Fasciola* antibodies. The high sensitivity of the SeroFluke test was at first glance surprising, since the sensitivity of LFIAs designed to be read by the naked eye is often lower than the equivalent laboratory-based ELISA tests [41]. Moreover, this may be even more surprising if we take into account that the antigen used in the MM3-SERO ELISA includes several L-type cathepsins (L1 and L2) from *Fasciola* [29], [31], whereas the SeroFluke test was constructed with a single-labelled artificially-refolded rpCL1 from *F. hepatica*, lacking some of the conformational epitopes that are present in mature cathepsins from this trematode [31]. Nevertheless, this may be explained by considering that positive sera from infected humans predominantly recognized the MM3 epitope present in the rpCL1, as revealed by ELISA inhibition studies in our laboratory (unpublished results). Thus, while in the MM3-SERO ELISA, the MM3-recognized epitope on the cathepsins is blocked by mAb MM3 during the process of antigen capture, in the SeroFluke test this epitope is available to react with human antibodies. The special preference for the MM3-epitope of the antibodies present in serum from infected humans may also explain the maximal specificity obtained in the present study, in which all patients infected with other related or unrelated parasites tested negative (Table 1). It is also possible that, in addition to the immunodominant MM3 epitope, other epitopes not present in mature cathepsin L from *Fasciola* contribute positively to recognition of the antigen by human sera. This may be the case of the B-cell epitopes, which were described in the prosegment region of the *Fasciola* cathepsin L [42]. However, the relevance of such epitopes as targets of anti-*Fasciola* serum antibodies in humans has not yet been investigated.

With respect to the specificity of the LFIA, it should also be noted that sera from patients infected with other liver food-borne trematodes (e.g. *Clonorchis sinensis* and *Opisthorchis viverrini*) were not available for study, which prevented us from establishing whether these pathogens may provoke false positive results. Nevertheless, as indicated above, since most of the anti-*Fasciola* antibodies are directed to the specific MM3-recognized epitope, cross-reactions with these species are not expected. This supposition is also consistent with a previous study reporting no recognition of a cathepsin L from *Clonorchis sinensis* by sera from patients infected with *Fasciola hepatica* [43].

Another aspect of the SeroFluke test to be considered when interpreting the results is that for some patients with large amounts of anti-*Fasciola* antibodies, a decrease in the intensity of the control line may be observed. This is due to competition between anti-*Fasciola* antibodies and the mAb MM3 immobilized in the control line for the MM3 epitopes present on the rpCL1. Therefore, a decrease in the signal in the control line often indicates that the sera tested have a high concentration of anti-*Fasciola* antibodies, rather than a failure of the device (for example due to incomplete liquid migration). Since the SeroFluke test was designed for use without a plastic housing, interpretation of the results is straightforward, as liquid migration can be observed directly. This is even more evident when testing whole blood samples because the migration of haemoglobin clearly stains the absorbent pad red (see Fig. 4). Nevertheless, if interpretation were doubtful, the test could be repeated with a more diluted sample (e.g. 1/500–1/1000) which would lead to an increase in signal intensity in the control line. The presence of mAb MM3 in the control line, rather than any other anti-rpCL1 antibody, may be advantageous for its use by trained personnel in hospitals, as this design enables qualitative estimation of the concentration of anti-*Fasciola* antibodies in serum by comparing the intensity of colour in the positive and control lines.

In previous studies with the MM3-SERO test in sheep [27], we have observed that such a test can be used to detect infections by either *F. hepatica* or *F. gigantica*. In this study we did not have the opportunity to test sera from patients infected with the latter species. However, although experimental confirmation is required, on the basis of our previous experience with the MM3-SERO ELISA, it appears likely that the new SeroFluke test will also be able to be used to detect human infections by *F. gigantica*.

In summary, we present a new highly stable, sensitive, and specific LFIA for serodiagnosis of human fasciolosis. It is expected that such a test will be useful for hospitals in hypoendemic regions as well as in hyperendemic regions where POC testing is required. Although serological tests cannot differentiate between current and past infections, these methods are very useful for detecting early infections, which may be then confirmed with data from coprological tests, when the patent period of infection has been reached, or with other complementary data. The suitability of the SeroFluke test for detection of antibodies in animal species is also being investigated.
